# Predictive models for anti-tubercular molecules using machine learning on high-throughput biological screening datasets

**DOI:** 10.1186/1756-0500-4-504

**Published:** 2011-11-18

**Authors:** Vinita Periwal, Jinuraj K Rajappan, Abdul UC Jaleel, Vinod Scaria

**Affiliations:** 1 GN Ramachandran Knowledge Center for Genome Informatics, CSIR Institute of Genomics and Integrative Biology (CSIR-IGIB), Mall Road, Delhi - 110007, India; 2 Department of Cheminformatics, Malabar Christian College, Calicut - 673001, Kerala, India; 3 Open Source Drug Discovery Consortium, Council of Scientific and Industrial Research (CSIR), Anusandhan Bhavan, 2 Rafi Marg, Delhi 110001, India

## Abstract

**Background:**

Tuberculosis is a contagious disease caused by *Mycobacterium tuberculosis *(Mtb), affecting more than two billion people around the globe and is one of the major causes of morbidity and mortality in the developing world. Recent reports suggest that Mtb has been developing resistance to the widely used anti-tubercular drugs resulting in the emergence and spread of multi drug-resistant (MDR) and extensively drug-resistant (XDR) strains throughout the world. In view of this global epidemic, there is an urgent need to facilitate fast and efficient lead identification methodologies. Target based screening of large compound libraries has been widely used as a fast and efficient approach for lead identification, but is restricted by the knowledge about the target structure. Whole organism screens on the other hand are target-agnostic and have been now widely employed as an alternative for lead identification but they are limited by the time and cost involved in running the screens for large compound libraries. This could be possibly be circumvented by using computational approaches to prioritize molecules for screening programmes.

**Results:**

We utilized physicochemical properties of compounds to train four supervised classifiers (Naïve Bayes, Random Forest, J48 and SMO) on three publicly available bioassay screens of Mtb inhibitors and validated the robustness of the predictive models using various statistical measures.

**Conclusions:**

This study is a comprehensive analysis of high-throughput bioassay data for anti-tubercular activity and the application of machine learning approaches to create target-agnostic predictive models for anti-tubercular agents.

## Background

Tuberculosis (TB) remains one of the largest killer infectious disease infecting one-third of the world's population. The latest World Health Organization estimates show that nearly 1.7 million people died of TB in 2009 [1]. Immunocompromised individuals, particularly those infected with human immunodeficiency virus (HIV) and those on immunosuppressant therapy are at greater risk for infection with Mycobacterium tuberculosis. HIV and TB form a lethal combination, accelerating disease progression and causing severe morbidity and mortality [2]. In addition, emergence and spread of multidrug-resistant (MDR) and extensively drug-resistant (XDR) strains have become a major concern worldwide [3]. An estimated 150,000 deaths caused by MDR-TB occurred globally in 2008, with almost 50% of the cases originating from China and India [4]. The discovery of new molecules with anti-TB activity and with no cross-resistance to any existing drugs has been the immediate need to control this global epidemic.

A modern drug discovery program generally starts with target selection and/or screening of small molecules, followed by hit identification, hit-to-lead transition, lead optimization, and clinical candidate selection. Hit identification, occurs at an early stage of the whole process and has profound influence on the success of any drug discovery program. High throughput screening has been one of the mainstays in the identification of hits in a general drug discovery programme. High throughput screening strategy suffers from its many limitations - most importantly, the enormous cost and time spent in running the screens [5]. Virtual screening, or in-silico screening which is a computational analogue of high throughput screening, has been employed as an early stage, cost-effective strategy to prioritize molecules from large compound libraries for experimental screening [6]. Virtual screening in addition to being cheaper than its experimental counterpart could further benefit from the advances in hardware and software development, including faster processors, parallel computing and smarter and faster algorithms.

Machine learning (ML) techniques and specifically supervised learning methods have been recently adopted for virtual screening to assign nominal/numerical classifications in terms of activities [7-12]. A major focus of ML methods is to automatically learn to recognize complex patterns which classify sets from input data and to make intelligent decisions based on independent datasets [13]. Bioactivity data available from the many high throughput screens provide useful means to train machine learning classifiers as it contains binary i.e. active/inactive as well as numerical (for example IC50) values for classification of compounds. Previous studies have pointed to the usability of bioassay data available in public domain to build efficient classifiers [10]. The recent availability of a large amount of data on biological activities of molecules, especially derived from high throughput screens now enables us to create predictive computational models. Though ML methods have proved to be a valuable tool in rapid virtual screening of large molecular libraries, they have been seldomly applied in TB drug discovery programmes [14-17]. Our present study aims at developing a comprehensive and systematic approach with the aid of ML techniques to build binary classification models from high throughput whole-cell screens of anti-tubercular agents. These predictive models when applied to virtual screening of large compound libraries can identify new active scaffolds that can accelerate the Mtb drug discovery process.

## Results and Discussion

All the three datasets from PubChem used in the present study were confirmatory in nature. A large imbalance was observed in the datasets with fewer numbers of actives compared to the inactives. The files containing 179 descriptors generated with PowerMV were loaded into Weka, after processing as described in the materials and methods section (vide supra). The numbers of descriptors finally used were 155,153 and 151 for AID1626, AID1949 and AID1332 respectively. Approximately 15% descriptors were removed from each dataset (Additional file 1). Since not many descriptors were removed using the diversity filter, we assume the compounds present in active and inactive datasets are quite diverse.

All the classification experiments were done on Weka version 3.6. We started with an increased heap-size of 4 GB to handle out-of-memory exceptions for large datasets. Initial experiments were run using standard classifiers alone. For models, having a low FP rate with standard classifiers, cost sensitivity was introduced. Misclassification cost was increased for false negatives so as to stay in the upper limit of FP rate. All results reported are based on independent test set used for re-evaluation on the trained model. Misclassification cost used for FN for each AID set is presented in Table 1. Lowest misclassification cost setting was required for Naive Bayes which was also the fastest building model.

**Table 1:** Misclassification costs used for False Negative (FN)

*Pubchem Assay ID*	*Naive Bayes*	*Random Forest*	*SMO*	*J48*
**AID1626**	9.5	6270	65	300
**AID1949**	8	2250	42	200
**AID1332**	None*	15	4	11

**Figure 1:**
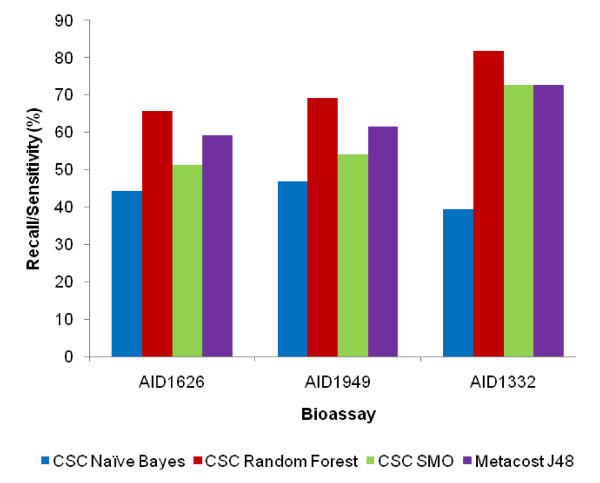
**Sensitivity Plot**. Plot of Sensitivity of each classifier for each dataset.

**Figure 2:**
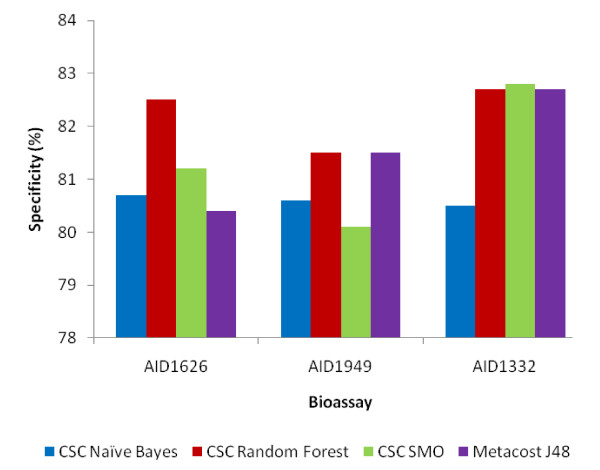
**Specificity Plot**. Plot of Specificity of each classifier for each dataset.

Since a number of models were trained on each dataset using different cost settings, best models of each dataset in each classifier category were selected based on various statistical measurements (Table 2) that were used to assess the performance of the models. All models were generated within controlled FP rate (i.e. 20%). The overall effectiveness of a classifier can be judged by the accuracy of the generated models. Almost all the models produced had accuracy near 80%. In order to make out the classifier's ability to efficiently identify actual positive and negative labels, a measure of Sensitivity (a.k.a. Recall-rate) and Specificity for each dataset was used respectively (Figure 1 and 2). An optimal prediction aims to achieve 100% sensitivity and specificity. As can be observed, the specificity of all the models was above 80% and the sensitivity varied from 50-80% for RF, J48 and SMO with NB being least sensitive. Random Forest appeared to be the most sensitive classifier for every dataset.

**Table 2:** Overall summary of the performance of the models

*Dataset*	Classifier*	*TP%*	*FP%*	*TN%*	*FN%*	*Accuracy*	*ROC area*	*BCR^#^*	*MCC^$^*
**AID1626**	CSCNB	44.4	19.3	80.7	55.6	80.32%	0.707	0.625	0.061
	CSCRF	65.7	17.5	82.5	34.3	82.36%	0.823	0.741	0.122
	CSCSMO	51.2	18.8	81.2	48.8	80.88%	0.750	0.662	0.080
	MetaCostJ48	59.3	19.6	80.4	40.7	80.24%	0.690	0.698	0.097
**AID1949**	CSCNB	46.9	19.4	80.6	53.1	80.04%	0.712	0.637	0.080
	CSCRF	69.2	18.5	81.5	30.8	81.25%	0.825	0.753	0.162
	CSCSMO	54.1	19.9	80.1	45.9	79.63%	0.744	0.671	0.107
	MetaCostJ48	61.6	18.5	81.5	38.4	81.17%	0.718	0.715	0.138
**AID1332**	NB	39.4	19.5	80.5	60.6	74.31%	0.686	0.599	0.171
	CSCRF	81.8	17.3	82.7	18.2	82.57%	0.876	0.822	0.521
	CSCSMO	72.7	16.2	83.8	27.3	82.11%	0.806	0.782	0.469
	MetaCostJ48	72.7	17.3	82.7	27.3	81.19%	0.762	0.777	0.454

* CSC denotes CostSensitiveClassifier, # BCR - Balanced Classification Rate,

$ MCC - Matthews Correlation Coefficient

Owing to the presence of class imbalance problem in our datasets, accuracy alone may not turn out to be the real estimator of model robustness. So, another performance measure called Balanced Classification Rate (BCR) was calculated. BCR, also referred to as Balanced Accuracy, equally weights the errors within each class. It gives a more precise evaluation of the overall model effectiveness. As can be observed from Table 2, the balanced accuracy of all models is optimum with Random Forest clearly outperforming others.

**Figure 3:**
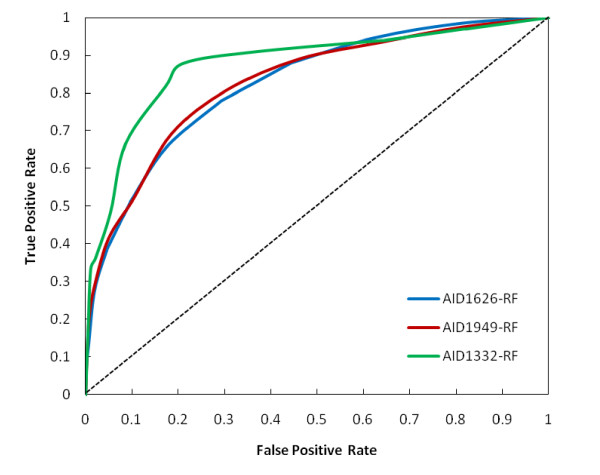
**ROC Plot**. ROC plot of Random Forest models of the three datasets.

For our highly imbalanced datasets accuracy and BCR turns out to be more reliable predictive measures as Meta-learning has been employed during model building. This increases our confidence in all the generated models. Best MCC values were obtained for Random Forest models (Table 2). A ROC curve analysis (Figure 3) of Random Forest models for all the three AIDs also revealed of the classifier's robustness and effectiveness. All the RF models had a significant Area under the Curve (AUC) of greater than 0.80. The ROC curves of other classifier's i.e. SMO, J48 and NB can be viewed in Additional file 2. The diagonal line (also referred to as line of no-discrimination) divides the ROC space. A completely random guess by the classifier would have resulted in points lying along the diagonal. As can be observed from the results, among all the four classifiers used Random Forest provides overall best classification for every AID followed by J48, SMO and NB. Thus we effectively propose Random Forest as the best classifier for currently studied Mycobacterium tuberculosis bioassay datasets that can be used to prioritize molecules for their selection during screening.

High dimensional nature of modeling tasks may raise the issue of over-fitting of the generated models and a number of measures are suggested to deal with this [18,19] thus it is better to cross-validate the performance of the models. Therefore, a cross-validation of best models generated in each category was also carried out by using other two AID sets as independent datasets (Table 3). This time the entire datasets were used as a test set. Satisfactory performance of all the models in cross-validation increased our reliability in the generated models. As expected all the models from every dataset performed equally well on a given test set. The higher accuracy of NB model of AID1332 as compared to RF, SMO and J48 models cannot be regarded effective enough as it was trained with standard classifier (i.e. without misclassification cost) thus it does not reflect a true measurement of performance of NB model. In our datasets, AID1626 had the largest number of compounds followed by AID1949 and AID1332. From the cross-validation results, we also deduce that training models on larger dataset result in more consistent predictions. As can be observed, cross-validation accuracy of AID1626 is more consistent than the other two datasets.

**Table 3:** Cross-validation of models on the three blinded data sets

*Test set*	*Model-set_AID1626 accuracy %*				*Model-set_AID1949 accuracy %*				*Model-set_AID1332 accuracy %*			
	**NB**	**RF**	**SMO**	**J48**	**NB**	**RF**	**SMO**	**J48**	**NB**	**RF**	**SMO**	**J48**

**AID1626**	-	-	-	-	82.7	82.7	83.3	80.0	78.1	66.3	66.1	61.8

**AID1949**	76.4	76.5	71.7	77.0	-	-	-	-	78.0	60.7	55.9	61.3

**AID1332**	75.5	79.9	74.5	74.6	73.0	78.0	74.2	66.7	-	-	-	-

## Conclusions

We have employed a systematic and comprehensive approach to build supervised classification based predictive models for anti-tubercular agents from publicly available bioassay datasets for in-vitro screens for Mtb inhibitors. In contrast with the conventional target-based screening approaches, these models are target-agnostic as they are based on whole-cell screening experiments. We have generated classification models for anti-tubercular agents with four commonly used state-of-the-art classifiers i.e. Naïve Bayes, Random Forest, J48 and SMO. Owing to the large class imbalance in datasets, introducing cost-sensitive meta-learning led to enhanced sensitivity, specificity and accuracy in the generated models. Though all the models produced had accuracy above 80%, the balanced accuracy (BCR) and AUC revealed a more precise and true picture of performance of the individual classifiers. An extensive analysis of binary classification statistical parameters showed that overall best classification for all three Mtb datasets was provided by Random Forest. Further, the cross-validation of best models against each of the other dataset also revealed a high predictive accuracy in the same range, substantiating the robustness of the model. These predictive models can now aid in the virtual screening of large molecular libraries to mine novel chemical scaffolds and thereby trigger an accelerated drug discovery for Mtb.

## Methods

### Data Source

**Figure 4:**
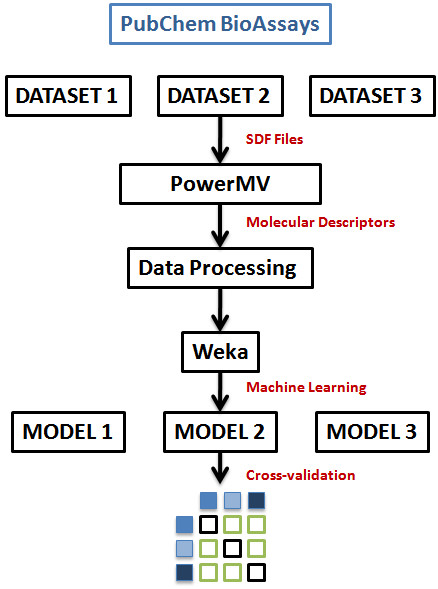
**Work flow**. Workflow for data collection, descriptor analysis, model building and validation.

Datasets for three confirmatory bioassay screens based on microplate Alamar blue assay for identifying inhibitors of Mycobacterium tuberculosis in 7H12 broth were available at PubChem database of National Center for Biotechnology Information (NCBI) [20]. These correspond to assay identifiers: AID1626, AID1949 and AID1332 [21,22]. All the three assays were conducted through the Tuberculosis Antimicrobial Acquisition and Coordinating Facility (TAACF) [23]. The total number of compounds tested in each assay along with the number of compounds identified as actives, inactives and inconclusive are enlisted in Additional file 3. Compounds that showed > 30% inhibition for at least one concentration in the anti-microbial activity dose response were defined as "Active". If the inhibition at all doses was < 30% in the Mtb assay, the compound was defined as "Inactive". Compounds with a percent inhibition > 80% but were not selected for follow up dose response were labeled "Inconclusive." Inconclusive compounds were not included in the training dataset to avoid uncertainty in predictive ability of the models. All the three confirmatory screens of inhibitors of Mycobacterium tuberculosis were downloaded in SDF formats. Their corresponding bioactivity data was also obtained from PubChem as a comma separated file. Figure 4 depicts the general flow of the strategy employed for data processing, analysis, model building and validation.

### Descriptor generation and dataset preparation

2D Molecular descriptors for all compounds were generated in the molecular visualization software PowerMV [24]. Owing to the large number of molecules in datasets AID1626 and AID1949 these files were split to smaller files using a Perl script SplitSDFiles available from MayaChemTools [25], prior to loading in PowerMV. Bioactivity values were appended to the data as class attribute (label: Outcome). A total of 179 descriptors were generated for each of the dataset. Details of various descriptors are provided in Additional file 4 and a comparative account of contribution of each descriptor to molecular properties of all compounds is summarized in Additional file 5. The bit-string fingerprint attributes having no variation i.e. the ones containing only 0's or 1's throughout the dataset were filtered out in order to reduce the dimensionality of the dataset. The datasets were ordered by class and a bespoke Perl script was used to randomly split them into two parts: 80% of the data was used as the training-cum-validation set and 20% of data as an independent test set. In Cross-validation, a number of folds n is specified. The dataset is randomly reordered and then split into n folds of equal size. In each iteration, one fold is used for testing and the other n-1 folds are used for training the classifier. The test results are collected and averaged over all folds. This gives the cross-validated estimate of the accuracy. Accounting for the computational complexity, a 5-fold cross-validation was performed on larger dataset and a 10-fold on smaller dataset.

### Machine Learning

We used the Weka data mining toolkit (version 3.6) for analysis of the data and classification experiments [26]. Weka incorporates comprehensive collection of machine learning algorithms for data mining tasks. It also incorporates tools for data pre-processing, classification, regression, clustering, association and visualization. The toolkit is also well-suited for developing new machine learning schemes.

### Classification Algorithms

Classification refers to an algorithmic procedure that attempts to assign each input value, a given set of classes. The classification process requires building a classifier (model) which is a mathematical function that assigns class (ex. active/inactive) labels to instances defined by a set of attributes (ex. descriptors). In the present study, we compared four state-of-the-art classifiers namely Naive Bayes, Random Forest, SMO and J48. The salient features of each of the classifiers are described below-

Naïve Bayes (NB) is based on Bayes rule [27]. The Naïve Bayes classifier learns from training dataset, the conditional probability of each attribute given the class label. This approach assumes that all descriptors are statistically independent (i.e. presence of one has no effect on the other) and therefore considers each of them individually. Bayes' theorem finds the probability of an event occurring given the probability of another event that has already occurred. The probability of a molecule to be in one or the other class is considered to be proportional to the ratio of members in each of the class that share the descriptor value. The overall probability of activity is computed by the product of the individual probabilities.

Random Forests (RF) are an ensemble of trees [28]. In order to grow these ensembles, random vectors are generated from the training set where a different set of descriptors is used to build each tree. Random vectors are drawn by randomly selecting a subset of features to grow each tree. After a large number of trees are grown, the vote for the most popular class is performed.

Sequential Minimal Optimization (SMO) is an implementation of Support Vector Machines (SVM) [29]. A SVM is a hyperplane that separates members of one class from members of another class (actives and inactives in this case) with maximum margin. Unlike SVM which requires solving a large quadratic programming (QP) optimization problem, SMO breaks this problem into smallest possible QP problems and solves them analytically. SMO is less costly in terms of the computation time and at the same time has the ability to handle large datasets compared to SVM, which makes it easy to implement it on very large datasets.

J48 is an implementation of C4.5 decision tree learner [30]. A decision tree model is constructed from root to leaves in a top-down fashion by choosing the most appropriate attribute at each node i.e. a decision node. A leaf indicates a class.

### Training Classifiers

Majority of the bioassay datasets are imbalanced where one class is represented by a large number of molecules while the other is not [31] which is observed in the present datasets also (Additional file 3). This class imbalance problem cannot be dealt successfully with standard data mining methods as most original classifiers assume equal weighting of the classes and that all misclassification errors cost equally. However, this assumption is not true in real world applications [32]. Cost-sensitive learning is often used to deal with datasets with very imbalanced class distribution. Introducing misclassification cost for standard classifiers makes them cost sensitive and provide them with the ability to predict a class that leads to the lowest expected cost [33]. Setting of the misclassification cost is almost always arbitrary as no general rule exists for it. It has previously been shown that misclassification cost depends more on the base classifier used rather than minority class ratio or number of attributes [10].

There are two ways to introduce misclassification cost in classifiers: one to design cost sensitive algorithms directly and the other is to use a wrapper that converts existing base classifiers into cost-sensitive ones. The later is called meta-learning [32]. In Weka Meta-learners are used to make the base classifiers cost sensitive. MetaCost [34] first uses bagging on decision trees to obtain reliable probability estimations of training examples, relabels the classes of training examples and then uses the relabeled training instances to build a cost-insensitive classifier. CostSensitiveClassifier [35], use a cost-insensitive algorithm to obtain the probability estimations of each test instance and then predicts the class label of the test examples.

For our datasets that are using a two class schema (i.e. active/inactive) cost sensitivity is introduced by using a 2 × 2 (for binary classification) cost matrix. The four sections of a cost matrix can be read as: True Positives (TP) - actives classified as actives; False Positives (FP) - inactives classified as actives; True Negatives (TN) - inactives classified as inactives; False Negatives (FN) - actives classified as inactives. One of the key point considered during development of the classifiers is that % of false negatives is more important than % of false positives for compound selection. To attain this, one could minimize the number of false negatives at the expense of increasing the false positive. Increasing misclassification for false negatives would lead to increase in both false positives and true positives. The % of false positives can easily be kept under check by setting an upper limit on FP rate. In this case the limit of FP rate was set to a maximum of 20%. The misclassification cost for false negatives could then be increased until this limit is achieved. Cases where standard classifiers were producing this result cost-sensitivity were not used and only default classifiers were used.

Default Weka options were used for building NB and RF models whereas in J48 unpruned tree option and in SMO buildlogisticmodels option was employed. For J48, MetaCost is used as it works better for unpruned trees. It treats the underlying classifier as a black box requiring no knowledge of its functioning. With NB, RF and SMO the standard CostSensitiveClassifier was used.

### Model Performance Evaluators

Various statistical binary classification performance measures were used to evaluate the results. A True Positive Rate (TPR) is the proportion of actual positives which are predicted positives (TP/TP+FN). False Positive rate (FPR) is ratio of predicted false actives to actual number of inactives (i.e. FP/FP+TN). Accuracy indicates proximity of measurement of results to the true value. It can be calculated as (TP+TN/TP+TN+FP+FN). Sensitivity (TP/TP+FN) relates to the test's ability to identify positive results whereas Specificity (TN/TN+FP) relates to the test's ability to identify negative results. A test with high sensitivity and specificity has a low error rate. A Balanced Classification Rate (BCR) (0.5*(sensitivity+specificity)) defined as mean of sensitivity and specificity gives a combined criteria of measurement that gives a balanced accuracy for unbalanced datasets. In addition to BCR, Matthews correlation coefficient (MCC) is also employed to judge performance of unbalanced datasets. Its value ranges from -1 to +1. A Receiver Operating Characteristic (ROC) curve is a graphical plot of TPR vs. FPR for a binary classification system. ROC space is defined by FPR and TPR on X and Y axes respectively. The Area under Curve (AUC) value reported by a ROC is equal to the probability that a classifier will rank a randomly chosen positive instance higher than a randomly chosen negative one.

## Competing interests

The authors declare that they have no competing interests.

## Authors' contributions

VS and AUCJ planned the project and methodology with VP who performed the data analysis. JKR supported the analysis. Members of the OSDDC were involved in scientific discussions. All authors contributed to the manuscript writing. All authors have read and approved the manuscript.

## Supplementary Material

Additional file 1 **List of descriptors before and after data processing**. Microsoft DOC file containing a table on detailed list of descriptors before and after data processing for all the three datasets Click here for file

Additional file 2 **ROC plot of SMO, J48 and NB**. Microsoft DOC file containing ROC graphs of SMO, J48 and NB Click here for file

Additional file 3 **Dataset details**. Microsoft DOC file containing a table on number of compounds in each dataset and their minority class ratios used in present analysis. Click here for file

Additional file 4 **List of descriptors**. Microsoft DOC file enlisting the descriptive account of various descriptors calculated for each dataset using PowerMV [22] Click here for file

Additional file 5 **Comparative account of molecular descriptors**. Contribution of each descriptor to molecular properties of all compounds Click here for file
