# Supplementary material for: Predictive models for anti-tubercular molecules using machine learning on high-throughput biological screening datasets
Source: BMC Res Notes. 2011 Nov 18;4:504. doi: 10.1186/1756-0500-4-504 (PMC3228709; doi:10.1186/1756-0500-4-504)
Supplement: Additional file 1 — List of descriptors before and after data processing. Microsoft DOC file containing a table on detailed list of descriptors before and after data processing for all the three datasets [file 1756-0500-4-504-S1.DOC]

**Additional Table** Detailed list of descriptors before and after data processing for each dataset

| Descriptor category* | Descriptors before data processing  (179) | Descriptors removed after data processing | | |
| --- | --- | --- | --- | --- |
|  |  | AID1626 | AID1949 | AID1332 |
| 1.Pharmacophore fingerprints (147) | NEG_01_NEG – NEG_07_NEG  NEG_01_POS – NEG_07_POS  NEG_01_HBD – NEG_07_HBD  NEG_01_HBA – NEG_07_HBA  NEG_01_ARC – NEG_07_ARC  NEG_01_HYP – NEG_07_HYP  POS_01_POS – POS_07_POS  POS_01_HBD – POS_07_HBD  POS_01_HBA – POS_07_HBA  POS_01_ARC – POS_07_ARC  POS_01_HYP – POS_07_HYP  HBD_01_HBD – HBD_07_HBD  HBD_01_HBA – HBD_07_HBA  HBD_01_ARC – HBD_07_ARC  HBD_01_HYP – HBD_07_HYP  HBA_01_HBA – HBA_07_HBA  HBA_01_ARC – HBA_07_ARC  HBA_01_HYP – HBA_07_HYP  HYP_01_HYP – HYP_07_HYP | NEG_01_POS  NEG_02_POS  NEG_01_HBA  NEG_02_HBA  NEG_01_ARC  NEG_01_HYP  POS_01_POS  POS_02_POS  POS_01_HBD  POS_01_HBA  POS_02_HBA  POS_01_ARC  POS_01_HYP  HBD_01_HBD  HBD_02_HBD  HBD_01_HBA  HBD_02_HBA  HBD_01_ARC  HBA_01_HBA  HBA_02_HBA  HBA_01_ARC  HBA_02_ARC  HBA_01_HYP  ARC_01_HYP | NEG_01_POS  NEG_02_POS  NEG_01_HBD  NEG_02_HBD  NEG_01_HBA  NEG_02_HBA  NEG_01_ARC  NEG_01_HYP  POS_01_POS  POS_02_POS  POS_01_HBD  POS_02_HBD  POS_01_HBA  POS_02_HBA  POS_01_ARC  POS_01_HYP  HBD_01_HBD  HBD_02_HBD  HBD_01_HBA  HBD_02_HBA  HBD_01_ARC  HBA_01_HBA  HBA_02_HBA  HBA_01_ARC  HBA_01_HYP  ARC_01_HYP | NEG_06_NEG  NEG_01_POS  NEG_02_POS  NEG_04_POS  NEG_07_POS  NEG_01_HBA  NEG_02_HBA  NEG_01_ARC  NEG_01_HYP  POS_01_POS  POS_02_POS  POS_01_HBD  POS_01_HBA  POS_02_HBA  POS_03_HBA  POS_01_ARC  POS_01_HYP  HBD_01_HBD  HBD_02_HBD  HBD_01_HBA  HBD_02_HBA  HBD_01_HYP  HBA_01_HBA  HBA_02_HBA  HBA_01_ARC  HBA_02_ARC  HBA_01_HYP  ARC_01_HYP |
| 2.Weighted Burden  Number (24) | WBN_GC_L_0.25  WBN_GC_H_0.25  WBN_GC_L_0.50  WBN_GC_H_0.50  WBN_GC_L_0.75  WBN_GC_H_0.75  WBN_GC_L_1.00  WBN_GC_H_1.00  WBN_EN_L_0.25  WBN_EN_H_0.25  WBN_EN_L_0.50  WBN_EN_H_0.50  WBN_EN_L_0.75  WBN_EN_H_0.75  WBN_EN_L_1.00  WBN_EN_H_1.00  WBN_LP_L_0.25  WBN_LP_H_0.25  WBN_LP_L_0.50  WBN_LP_H_0.50  WBN_LP_L_0.75  WBN_LP_H_0.75  WBN_LP_L_1.00  WBN_LP_H_1.00 | None | None | None |
| 3.Properties (8) | XLogP, PSA, NumRot, NumHBA, NumHBD, MW, BBB, BadGroup | None | None | None |

* Values in brackets depict the number of descriptors calculated in each category
