# Supplementary figures and images for: Predictive models for anti-tubercular molecules using machine learning on high-throughput biological screening datasets
Source: BMC Res Notes. 2011 Nov 18;4:504. doi: 10.1186/1756-0500-4-504 (PMC3228709; doi:10.1186/1756-0500-4-504)

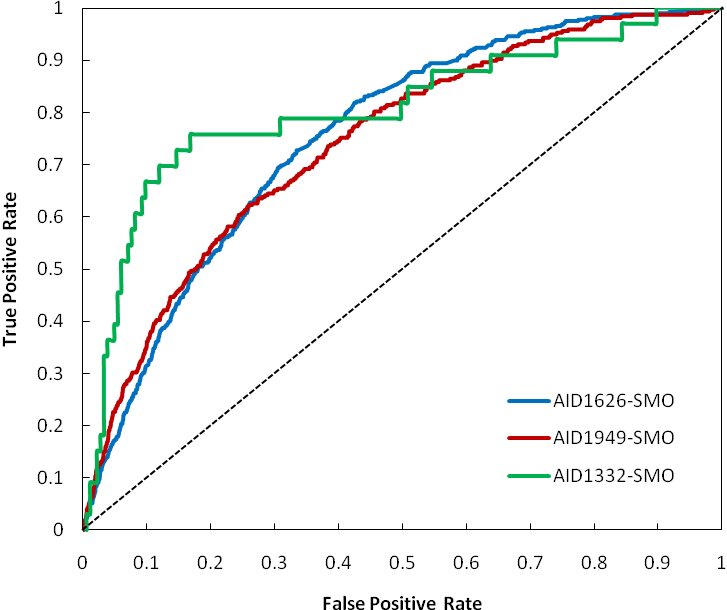


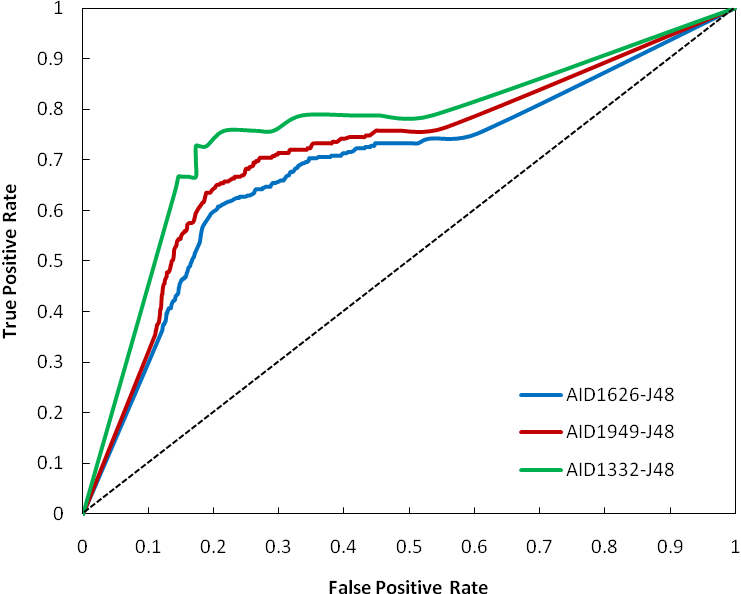


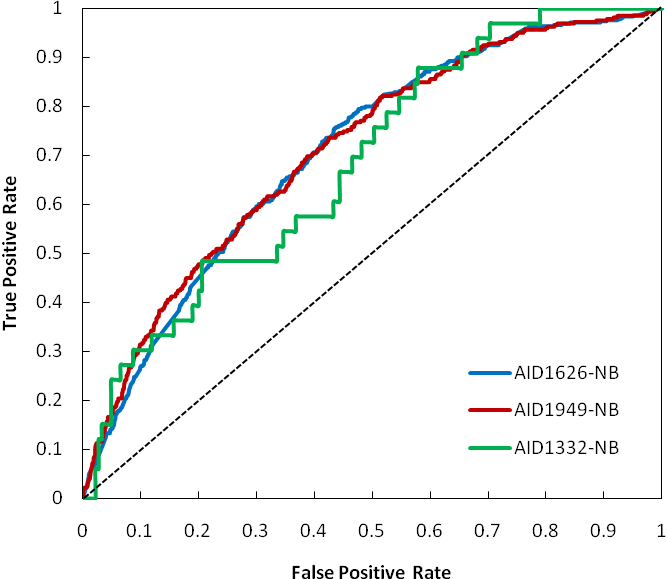

Supplement: Additional file 2 — ROC plot of SMO, J48 and NB. Microsoft DOC file containing ROC graphs of SMO, J48 and NB [file 1756-0500-4-504-S2.DOC]
