# Supplementary material for: Predictive models for anti-tubercular molecules using machine learning on high-throughput biological screening datasets
Source: BMC Res Notes. 2011 Nov 18;4:504. doi: 10.1186/1756-0500-4-504 (PMC3228709; doi:10.1186/1756-0500-4-504)
Supplement: Additional file 3 — Dataset details. Microsoft DOC file containing a table on number of compounds in each dataset and their minority class ratios used in present analysis. [file 1756-0500-4-504-S3.DOC]

**Additional Table** Details of datasets used in present analysis

| *PubChem Assay ID* | *Number of Actives* | *Number of Inactives* | *Number of Inconclusives* | *Total Number of Molecules* | *Minority class %* |
| --- | --- | --- | --- | --- | --- |
| AID1626 | 2044 | 209569 | 3502 | 215107 | 0.9 |
| AID1949 | 1594 | 95916 | 3175 | 100685 | 1.6 |
| AID1332 | 166 | 927 | 24 | 1117 | 17.9 |
