# Supplementary material for: Predictive models for anti-tubercular molecules using machine learning on high-throughput biological screening datasets
Source: BMC Res Notes. 2011 Nov 18;4:504. doi: 10.1186/1756-0500-4-504 (PMC3228709; doi:10.1186/1756-0500-4-504)
Supplement: Additional file 4 — List of descriptors. Microsoft DOC file enlisting the descriptive account of various descriptors calculated for each dataset using PowerMV [22] [file 1756-0500-4-504-S4.DOC]

**Additional Table** Various descriptors calculated for each dataset

| Descriptor category* | Description | Division |
| --- | --- | --- |
| 1.Pharmacophore fingerprints (147) | based on bioisosteric principles | Atom/group bearing formal negative charge  Atom/group bearing formal positive charge  Hydrogen-bond donor  Hydrogen-bond acceptor  Aromatic center  Hydrophobic center |
| 2.Weighted Burden  Number (24) | based on burden  connectivity matrix | Electro negativity, Gasteiger partial charge or atomic lipophilicity, XlogP |
| 3.Properties (8) | useful for judging the drug-like nature of a molecule | XlogP, polar surface area,  number of rotatable bonds, H-bond donors, H-bond acceptors, molecular weight, blood-brain indicator and bad group indicator |

* Values in brackets depict the number of descriptors calculated in each category.
